# Ultrasound-guided lateral kidney biopsy in patients unsuitable for the prone position

**DOI:** 10.1093/ckj/sfag038

**Published:** 2026-02-12

**Authors:** Yuki Oba, Hisashi Kamido, Masatoshi Yoshimoto, Shigekazu Kurihara, Daisuke Ikuma, Hiroki Mizuno, Masayuki Yamanouchi, Tatsuya Suwabe, Yoshifumi Ubara, Kei Kono, Kenichi Ohashi, Naoki Sawa

**Affiliations:** Nephrology Center and Department of Rheumatology, Toranomon Hospital Kajigaya, Kanagawa, Japan; Department of Human Pathology, Graduate School of Medical and Dental Sciences, Institute of Science Tokyo, Tokyo, Japan; Nephrology Center and Department of Rheumatology, Toranomon Hospital Kajigaya, Kanagawa, Japan; Nephrology Center and Department of Rheumatology, Toranomon Hospital Kajigaya, Kanagawa, Japan; Nephrology Center and Department of Rheumatology, Toranomon Hospital Kajigaya, Kanagawa, Japan; Nephrology Center and Department of Rheumatology, Toranomon Hospital Kajigaya, Kanagawa, Japan; Nephrology Center and Department of Rheumatology, Toranomon Hospital Kajigaya, Kanagawa, Japan; Nephrology Center and Department of Rheumatology, Toranomon Hospital Kajigaya, Kanagawa, Japan; Nephrology Center and Department of Rheumatology, Toranomon Hospital Kajigaya, Kanagawa, Japan; Nephrology Center and Department of Rheumatology, Toranomon Hospital Kajigaya, Kanagawa, Japan; Department of Pathology, Toranomon Hospital, Tokyo, Japan; Department of Human Pathology, Graduate School of Medical and Dental Sciences, Institute of Science Tokyo, Tokyo, Japan; Department of Pathology, Toranomon Hospital, Tokyo, Japan; Nephrology Center and Department of Rheumatology, Toranomon Hospital Kajigaya, Kanagawa, Japan

**Keywords:** abdominal aortic aneurysm, kidney biopsy, lateral position, organomegaly, TAFRO syndrome

## Abstract

**Background:**

Kidney biopsy is the gold standard diagnostic technique for nephrologists. Appropriate patient positioning facilitates physiological stability and access to target anatomy. Factors such as patient weight, size and medical history, including respiratory or circulatory disorders, should guide position selection. However, most kidney biopsies are performed exclusively in the prone position and procedures are often deferred when this is not feasible. Although lateral kidney biopsy has been reported, it remains uncommon and its applications are not well established. We aimed to demonstrate the safety and utility of lateral kidney biopsies.

**Methods:**

We retrospectively reviewed patients who underwent lateral kidney biopsy at Toranomon Hospital Kajigaya between October 2015 and February 2025. Vital signs and blood test results pre- and post-biopsy were analysed.

**Results:**

Twenty-five patients underwent lateral kidney biopsies; six had abdominal aortic aneurysms (AAAs), 12 had respiratory distress (7 with massive ascites or pleural effusions, 2 with obesity, 2 with organomegaly and 1 with pericardial effusion), 6 had major joint pain (4 thin, 1 with scoliosis and 1 with arthritis) and 1 had stoma. All patients were histologically diagnosed and received appropriate treatment. Vital signs remained stable and only one patient with splenomegaly experienced a bleeding complication, which was not readily predictable and unlikely related to biopsy position.

**Conclusions:**

Lateral kidney biopsy offers a viable alternative for the accurate diagnosis of kidney diseases in patients in whom the prone position is challenging, including those with AAA, anasarca-induced respiratory distress or significant joint pain due to body habitus.

## Graphical Abstract

**Graphical Abstract:**
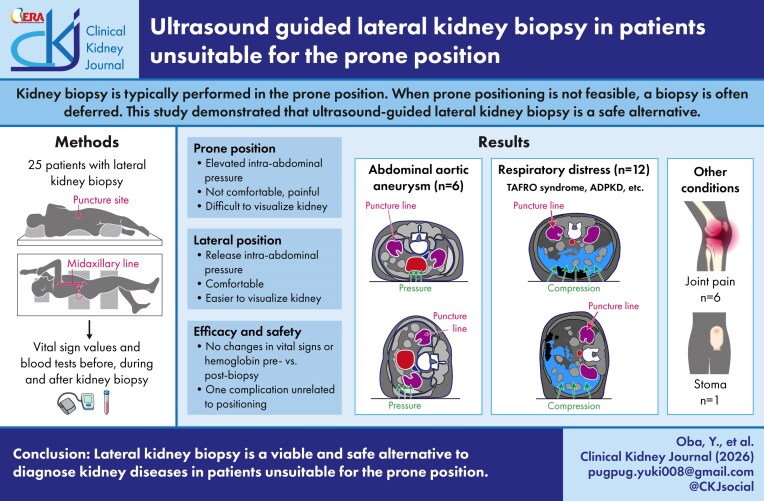

KEY LEARNING POINTS **What was known:**

- Most kidney biopsies are performed in the prone position; however, this is not feasible in some cases.
- Previous reports have introduced lateral kidney biopsy for pregnant women, severely obese patients and those with respiratory diseases.
- However, it remains uncommon and other indications have not been explored.

**This study adds:**

- We suggest that lateral kidney biopsy may be a feasible option with an acceptable safety profile even in selected challenging cases, including those with abdominal aortic aneurysm, anasarca-induced respiratory distress and significant joint pain due to body habitus.

**Potential impact:**

- Lateral kidney biopsy is a viable alternative method for the accurate diagnosis of kidney diseases in patients who cannot tolerate the prone position.
- This approach helps to prevent missed treatment opportunities and mitigates the potential negative impact of deferred biopsy on renal prognosis.

## Introduction

Appropriate patient positioning facilitates physiological stability and access to certain anatomical locations during surgical procedures. Multiple factors should be considered when selecting the patient’s position, including patient weight, size and medical history (including respiratory and circulatory disorders). However, kidney biopsies are mostly performed in the prone position [1]. Since Iversen and Brun [2] introduced percutaneous kidney biopsy in 1951, the procedure has become indispensable for diagnosis, prognosis and therapy. Kark and Muehrcke [3] standardized the prone approach position in 1954, and subsequent technical refinements, including ultrasound guidance, have reinforced its widespread adoption. In Japan, Kinoshita [4] established a kidney biopsy procedure at Niigata University in 1954, initially using the sitting position; however, advances in needle technology and imaging led to the establishment of the prone, ultrasound-guided technique as standard practice. Although the Japanese Kidney Biopsy Guidebook was updated in 2020, following a nationwide survey [5], patient positioning was not addressed. Routine use of the prone position has limited critical evaluation of alternative biopsy approaches.

Some patients may have difficulty undergoing biopsies in the prone position for various reasons. In addition, abdominal compression in the prone position can trigger vagal reflexes, resulting in hypotension and discomfort. In such cases, deferring kidney biopsy because of positioning limitations may lead to missed treatment opportunities and adversely affect renal prognosis. Previous studies have introduced lateral kidney biopsies [6–8], with demonstrated efficacy and safety in pregnant women, severely obese patients and patients with respiratory diseases. However, lateral kidney biopsy remains uncommon and its applicability to other clinical settings has not been widely explored. Here we aimed to demonstrate other situations where lateral kidney biopsy is useful, discuss its safety and present it as a feasible alternative approach.

## Materials and methods

### Patient selection

We collected data on patients who underwent lateral kidney biopsy at Toranomon Hospital Kajigaya between October 2015 and February 2025 from the kidney biopsy registry. The decision to perform the biopsy in the lateral position was made at the discretion of the physician. We extracted vital signs before, during and after kidney biopsy from electronic medical records. Blood tests were routinely conducted 1 day before and 1 day after the biopsy, with the values collected as pre- and post-procedure measurements.

This study was performed in accordance with the Declaration of Helsinki and its revisions and was approved by the Research Ethics Committee of Toranomon Hospital (approval number 2604-B). The requirement for informed consent was waived because of the retrospective nature of the study.

### Lateral kidney biopsy

**Figure 1:**
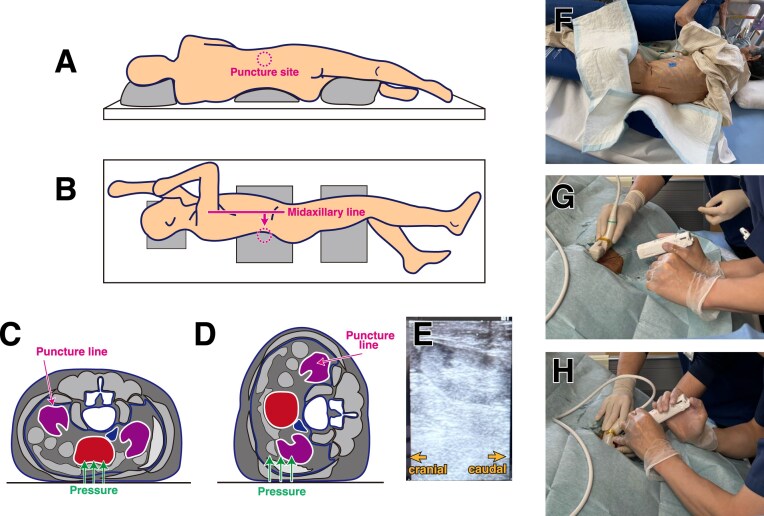
Lateral kidney biopsy procedure. **(A, B)** Cushions were placed under the patient to ensure comfort, extend the flank and enhance visualization of the kidney on ultrasound. The planned puncture site was marked posterior to the midaxillary line (circle). **(C, D)** Axial schematic images of patients with AAAs. The prone position exerts greater direct pressure on the AAA, whereas the lateral position reduces this pressure. **(E)** Ultrasound image during the lateral kidney biopsy for the patient with an AAA at the puncture site. The left side of the image is cranial and the right side is caudal. No structures obstructed the path from the body surface to the kidney and no aortic aneurysm was observed below the kidney. **(F)** An example of actual patient positioning. **(G, H)** Echo-guided biopsy was performed with the needle inserted perpendicular to the patient’s body.

Figure 1 and the supplemental video illustrate the lateral kidney biopsy procedure. The lateral position was selected when minimising abdominal pressure was considered necessary [e.g. abdominal aortic aneurysm (AAA) or stoma] or when patients reported difficulty tolerating the prone position owing to dyspnea or discomfort. Patient comfort was ensured while positioning them to allow for optimal ultrasound visualization of the kidney. For example, patients were instructed to hold onto bedrails or place cushions between their legs. In some cases, a cushion was placed beneath the patient to extend the flank and enhance ultrasound visualization of the kidney (Fig. 1A). The puncture site was selected posterior to the midaxillary line, as the kidneys are retroperitoneal organs (Fig. 1B). Figure 1C and D show axial images of patients with AAA. As illustrated in Fig. 1C, the prone position exerted greater direct pressure on the AAA, whereas the lateral position reduced this pressure (Fig. 1D). Figure 1E shows the ultrasound image obtained during the procedure in a patient with an AAA. No aneurysm was observed below the kidney. Figure 1F–H illustrate the biopsy procedure. Kidney biopsies were performed using an automatic, spring-loaded 16-gauge core biopsy needle gun (Acecut; Create Medic, Yokohama, Japan) identical to that used for kidney biopsies in the prone position. Provided that both the kidney and needle were visible on ultrasound, the needle trajectory was not restricted. In almost all cases (Fig. 1G and H), the needle was inserted perpendicular to the patient’s backand parallel to the floor. After the biopsy, manual compression was applied in an anterior–posterior direction using both hands for 15 min. Desmopressin was not administered before the kidney biopsy. To prevent bleeding complications, intravenous carbazochrome sodium sulfonate and tranexamic acid were administered during the procedure, followed by oral administration of both agents for 2 days post-biopsy. These haemostatic agents are commonly used in clinical practice in Japan [5].

### Statistical analysis

Baseline patient characteristics were summarised using means with interquartile ranges (IQRs) for continuous variables and percentages for categorical variables. The clinical findings or comorbidities, differential diagnoses and histological diagnoses were summarised in a table. The Wilcoxon signed-rank test was used to assess significant changes in vital signs and progression of anemia before, during and after kidney biopsy. All statistical analyses were performed using Stata/MP version 15.0. (StataCorp, College Station, TX, USA).

## Results

Among the 2076 kidney biopsies, 25 patients (1.20%) underwent biopsy in the lateral position. Table 1 summarizes their characteristics. Twenty patients were male with an average age of 66.6 years. Sixteen patients underwent right kidney biopsy in the left lateral position. No patient received antiplatelet or anticoagulant therapy at the time of kidney biopsy. The patient’s position remained unchanged during the procedure. In patients 1–18 and patient 25, the lateral position was selected from the outset. However, for patients with joint pain (patients 19–24), we initially attempted the prone position and switched to the lateral position when they reported severe pain in the prone position. The average number of biopsy cores was 4.2. The average total number of glomeruli was 38.6, with a mean of 9.5 glomeruli per core, which was sufficient for diagnosis.

**Table 1:** Characteristics of all patients who underwent lateral kidney biopsy.

Patient	1	2	3	4	5	6	7	8	9	10	11	12	13	14	15	16	17	18	19	20	21	22	23	24	25
	Abdominal aortic aneurysm						Respiratory distress												Joint pain						Other
	Diameter (mm)						Massive ascites and/or pleural effusions									Organomegaly									
Categories of reasons for lateral kidney biopsy	25	29	46	53	54	63	TAFRO syndrome			Nephrotic syndrome		Unknown cause		Obesity		ADPKD	TAFRO	Pericardial effusions	Thinness				Scoliosis	Arthritis	Stoma
Characteristics																									
Age (years)	69	82	66	77	76	75	77	35	52	46	83	64	53	81	71	71	39	74	45	48	72	84	79	76	69
Sex	M	M	M	M	M	M	M	M	M	M	M	M	F	F	M	F	M	M	M	M	M	F	M	M	F
BMI (kg/m^2^)	23.8	22.4	23.2	24.4	18.7	22.4	22.7	26.1	28.2	32.2	25.7	26.8	18.7	32.8	24.9	19.3	25.4	25.8	20.0	16.7	10.9	14.1	20.7	20.4	20.5
Laboratory data																									
Creatinine (mg/dL)	1.68	2.59	1.55	1.14	3.95	1.12	5.87	6.28	4.24	1.70	2.76	7.18	0.50	0.80	1.33	2.67	1.28	2.26	1.01	2.42	1.30	2.44	1.25	3.86	0.73
Cystatin C (mg/L)													1.21	1.16			1.83	2.7							
eGFR_cre_ (ml/min/1.73 m^2^)	32.8	19.3	36.1	48.3	12.5	49.6	8.00	9.50	12.9	36.2	18.0	6.80	97.9	51.8	41.8	14.4	51.7	23.1	64.4	23.4	42.7	15.1	43.4	12.8	60.0
eGFR_cys_ (ml/min/1.73 m^2^)													56.3	52.0			40.1	20.2							
UPCR (g/g creatinine)	4.24	7.25	5.73	0.45	7.45	0.07	0.55	0.87	0.35	5.38	10.99	1.66	1.94	1.07	0.38	2.52	0.05	0.04	0.66	7.11	3.35	2.17	0.69	2.60	0.65
Occult blood (/HPF)	>30	1–4	1–4	1–4	5–9	10–30	>50	>30	>30	5–9	>30	>30	5–9	1–4	1–4	10–30	1–4	1–4	10–30	10–30	10–30	<1	1–4	>30	1–4
Kidney size (mm)																									
Right kidney	80. 3	72.4	92.5	95.9	92.4	96.4	88.6	92.7	104.3	120.0	85.2	105.0	103.5	89.6	91.0	98.9	98.5	61.6	94.0	100.9	78.0	74.5	91.0	80.1	91.2
Left kidney	75.7	86.2	106.2	83.7	112.1	102.1	90.1	105.5	109.8	125.0	81.4	105.0	120.7	60.0	85.0	114.0	96.3	71.8	109.5	77.2	81.0	80.8	96.9	90.5	112.4
Punctured kidney	R	L	R	R	R	L	R	R	L	L	R	R	R	R	R	R	R	L	R	L	L	L	R	L	R
Punctures, *n*	6	4	4	4	4	5	8	5	7	5	6	3	6	5	4	5	6	8	4	6	7	7	5	15	5
Cores, *n*	5	4	4	3	3	5	8	4	5	5	4	2	4	3	4	3	6	5	3	2	4	4	3	7	4
Glomeruli, *n*	11	39	15	15	24	83	36	52	88	8	26	14	23	35	33	16	24	65	44	27	58	23	38	90	78
Glomeruli/core, *n*	2.2	9.8	3.8	5.0	8.0	16.6	4.5	13.0	17.6	1.6	6.5	7.0	5.8	11.7	8.3	5.3	4.0	13.0	14.7	13.5	14.5	5.8	12.7	12.9	19.5
Cortex/medulla, *n*	6/4	6/4	8/2	5/5	5/5	8/2	10/0	10/0	8/2	4/6	7/3	7/3	6/4	10/0	5/5	4/6	4/6	8/2	5/5	7/3	10/0	7/3	8/2	8/2	7/3
Antiplatelet or anticoagulant therapy	None	None	None	None	None	None	None	None	None	None	None	None	None	None	None	None	None	None	None	None	None	None	None	None	None

ADPKD: autosomal dominant polycystic kidney disease; F: female; M: male; eGFR: estimated glomerular filtration rate; HPF: high-power field; cre: creatinine; cys: cystatin; R: right; L: left

Six patients had an AAA, contraindicating the application of abdominal pressure. Twelve patients experienced respiratory distress while in the prone position. Seven patients had pleural effusion or ascites, two were obese, two exhibited organomegaly and one had pericardial effusion. Six patients experienced pain in the large joints. One patient underwent an abdominal colostomy. Table 1 shows their clinical characteristics and Table 2 shows their clinical findings or comorbidities, differential diagnoses and histological diagnoses.

**Table 2:** Clinical and histological diagnosis and treatments.

Patient	Clinical findings or comorbidities	Clinical differential diagnosis	Histological diagnosis	Treatment	Unexpected diagnosis	Change treatment plan
1	Diabetes mellitus with hematuria	Glomerulonephritis	Diabetic nephropathy, Tervaert class IIA	Supportive care	N	N
			Moderate to severe arteriosclerosis and arteriolosclerosis			
2	Hypogammaglobulinemia	Glomerulonephritis	Tubulointerstitial nephritis	Transferred to hematology and started chemotherapy	N	N
	M proteinemia	Cast nephropathy	Plasmacytic infiltration with light chain restriction			
3	Psoriasis, psoriatic arthritis, NS	Glomerulonephritis	MN with nephrosclerotic and FSGS changes	Adalimumab for psoriasis	N	Y
			Severe arteriosclerosis and moderate arteriolosclerosis			
4	Kidney impairment	IgAN	Nephrosclerosis	Supportive care	Y	Y
			Moderate arteriosclerosis and arteriolosclerosis			
5	NS	Diabetic nephropathy	IgG4-related tubulointerstitial nephritis	Glucocorticoids	Y	Y
	Diabetes mellitus		Severe arteriosclerosis			
6	MPA	Necrotizing glomerulonephritis	Minor glomerular abnormalities, mild IFTA	Avacopan and rituximab for MPA-induced interstitial pneumonia	Y	N
7	TAFRO syndrome	TMA	TMA	Glucocorticoids and tocilizumab	N	N
8	TAFRO syndrome	TMA	Diffuse endocapillary lesion with tubular vacuolization, TMA	Glucocorticoids and tocilizumab	N	N
9	TAFRO syndrome	TMA	TMA	Glucocorticoids and tocilizumab	N	N
10	Alcoholic liver cirrhosis	IgAN	IgAN (Oxford classification M0, E0, S1, T0, C1),	Supportive care with SGLT2i	N	N
		Diabetic nephropathy	Moderate arteriolosclerosis			
11	NS with M proteinemia	ANCA-negative vasculitis	FSGS, collapsing variant	Stopped glucocorticoid treatment and switched to supportive care	Y	Y
		MGRS	Moderate arteriosclerosis and moderate arteriolosclerosis			
12	Mixed phenotype acute leukemia, post-CBT	Transplant-associated TMA	Diabetic nephropathy, Tervaert class IIA	Supportive care	Y	Y
13	ANA positive (x5120)	Lupus nephritis	Minor glomerular abnormalities	Glucocorticoids for enteritis and cystitis	Y	N
	Lupus-like enteritis	Sjögren’s syndrome				
	Hydronephrosis caused by cystitis					
14	Rheumatoid arthritis	Obesity-related glomerulopathy	Secondary FSGS, perihilar variant	Supportive care	N	N
		Rheumatoid arthritis–related glomerulonephritis				
15	MPA, necrotizing glomerulonephritis	Worsening of MPA	Nephrosclerosis with focal crescent formation	Restart glucocorticoids	N	N
16	ADPKD with abnormal urinalysis	Glomerulonephritis	IgAN (Oxford classification M0, E0, S1, T1, C0)	Supportive care	N	N
	Recurrent cyst infection					
17	TAFRO syndrome	TMA	TMA, slightly IgA deposition	Glucocorticoids and tocilizumab	N	N
18	Kidney impairment with abnormal urinalysis	Glomerulonephritis	Nephrosclerosis	Supportive care	Y	Y
			Severe arteriosclerosis and arteriolosclerosis			
19	Kidney impairment with abnormal urinalysis	Glomerulonephritis	IgAN (Oxford classification M0, E0, S0, T0, C0)	Tonsillectomy and steroid pulse	N	N
20	FLT3-positive AML, post-CBT	Transplant-associated TMA	TMA	Glucocorticoids	N	N
			Severe IFTA, severe arteriosclerosis and arteriolosclerosis			
21	MPO-ANCA positive	MPO-AAV	Severe IFTA with moderate lymphocytic infiltration	Glucocorticoids	Y	N
			Severe arteriosclerosis			
22	Kidney impairment with abnormal urinalysis	Worsening of IgAN	Nephrosclerosis	Supportive care	Y	Y
	previously diagnosed IgAN	Secondary tubulointerstitial nephritis	severe IFTA with lymphocytic infiltration			
	Sjögren’s syndrome		severe arteriosclerosis and arteriolosclerosis			
23	ANA positive	Lupus nephritis	Mild mesangial thickening with focal mesangiolytic change	Supportive care	Y	Y
			Slight IgA deposition.			
			Mild arteriosclerosis and arteriolosclerosis			
24	Rapidly progressive glomerulonephritis with MPO-ANCA positivity	MPO-AAV	IgA nephropathy with nephrosclerosis and FSGS-changes	Glucocorticoids and avacopan	Y	N
			Severe arteriosclerosis and arteriolosclerosis			
25	Lupus nephritis IV-G(A/C)+V, 8 years ago with glucocorticoids taken 3.5 mg/day	Worsening of lupus nephritis	Lupus nephritis IV-G(A/C)+V	Start methylprednisolone pulse, add on tacrolimus and increased MMF	Y	N

NS: nephrotic syndrome; MN: membranous nephropathy; MPA: microscopic polyangiitis; IFTA: interstitial fibrosis and tubular atrophy; TMA: thrombotic microangiopathy; CBT: cord blood transplantation; FSGS: focal segmental glomerular sclerosis; AML: acute myeloid leukemia; MPO: myeloperoxidase; AAV: ANCA-associated vasculitis; MMF: mycophenolate mofetil; Y: yes, N: no.

### Patients with an AAA

**Figure 2:**
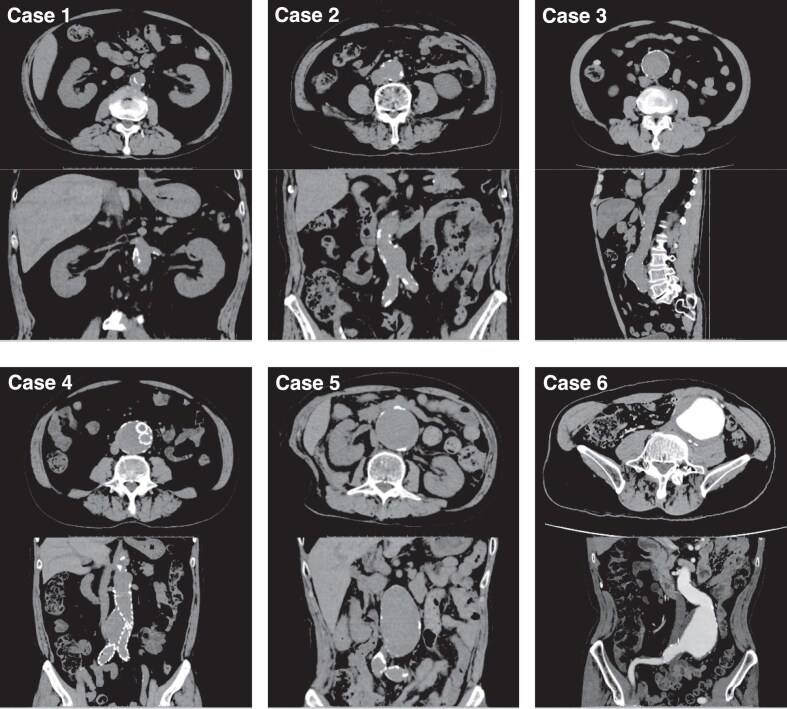
CT ima:ges of the patients with AAAs (patients 1–6).

Figure 2 shows the computed tomography (CT) images of six patients with an AAA. Patients 1–6 are arranged in ascending order of aneurysm diameter. Patient 1 had the smallest (24.6 mm in diameter) and it appeared more consistent with a dissection than an aneurysm, with vascular calcification separated from the vessel wall. Patient 4 involved post-stenting with a large stent diameter of 53 mm. In either case, many nephrologists would hesitate to perform kidney biopsies in the prone position to avoid exerting abdominal pressure.

### Patients with respiratory distress

**Figure 3:**
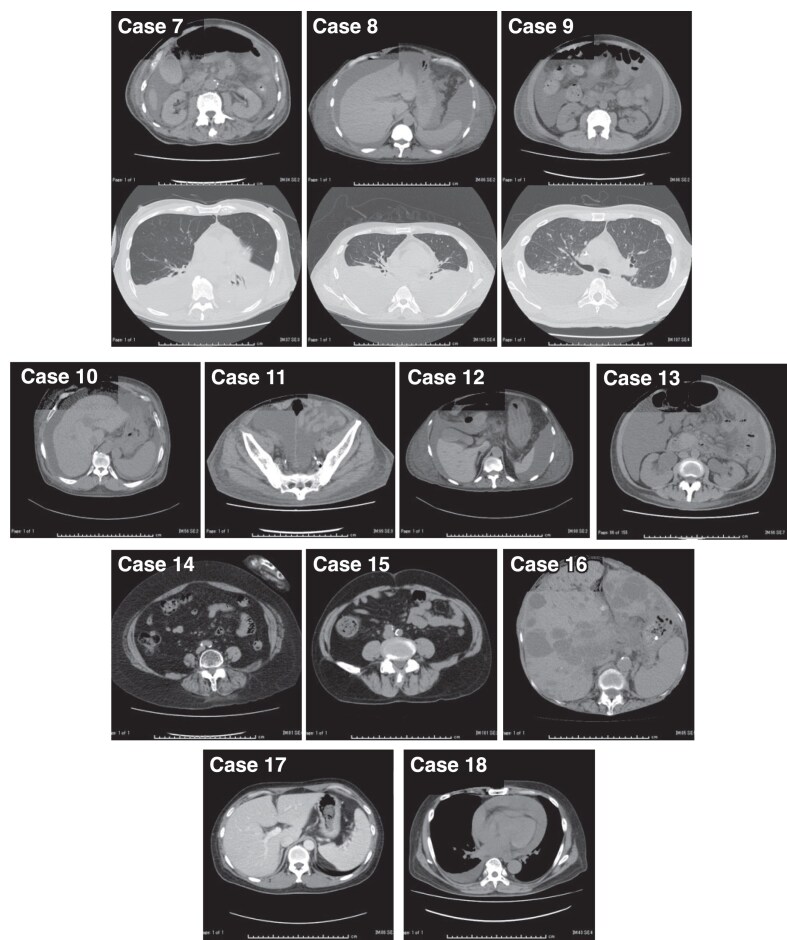
CT images of the patients with respiratory distress (patients 7–18). Patients 7–13 had massive ascites and pleural effusions. Patients 14 and 15 were obese. Patients 16 and 17 exhibited organomegaly and patient 18 had pericardial effusion.

Twelve patients experienced respiratory distress. Figure 3 shows their CT images. Seven patients had massive pleural effusion and ascites, with varied etiologies of anasarca. Three of these patients (patients 7–9) were diagnosed with thrombocytopenia, anasarca, fever, reticulin fibrosis/renal insufficiency and organomegaly (TAFRO) syndrome. Patient 7 had gained 15.8 kg in weight and required 3 L/min of oxygen through a nasal cannula. Patient 8 had gained 23.7 kg and required 8 L/min of oxygen via a reservoir mask. Patient 9 had gained 25.0 kg and required 10 L/min of oxygen via a reservoir mask. Kidney biopsies revealed glomerular endotheliosis, confirming the diagnosis of TAFRO syndrome and guiding treatment with glucocorticoids and tocilizumab. As the kidneys are retroperitoneal, ascites in the preperitoneal cavity did not interfere with kidney biopsies.

Two patients (patients 14 and 15) were obese, with distances from the body surface to the lower kidney pole on CT measuring 8 and 9 cm, respectively. Two patients (patients 16 and 17) exhibited organomegaly. Patient 16 had hepatomegaly due to autosomal dominant polycystic kidney disease and patient 17 had splenomegaly due to TAFRO syndrome. Patient 18 had a pleural effusion of unknown etiology.

### Patients with joint pain

**Figure 4:**
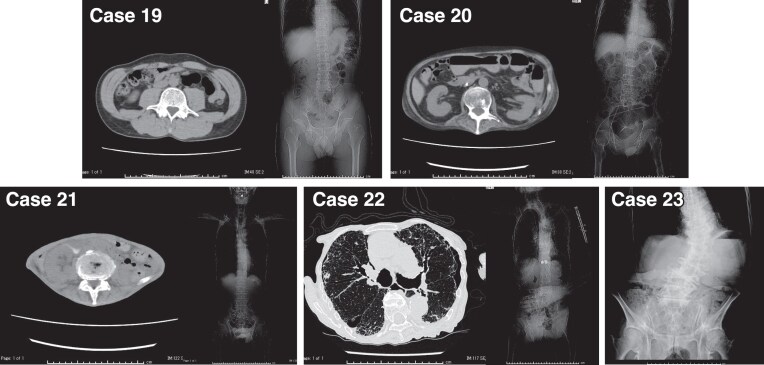
Radiographic images of the patients with major joint pain. Patients 19–22 were thin and patient 22 presented with interstitial pneumonia accompanied by pneumothorax. Patient 23 exhibited scoliosis.

Six patients (patients 19–24) experienced joint pain in the prone position, attributable to their physiques or underlying medical conditions. Four patients (patients 19–22) were thin, with a body mass index (BMI) of <20.0. One patient (patient 23) exhibited severe scoliosis, and the left kidney was difficult to observe on echography. Figure 4 shows images of their physiques. One patient (patient 24) presented with joint pain due to anti-nuclear cytoplasmic antibody (ANCA)-associated vasculitis and experienced pain when his/her bones touched the bed while in the prone position. The lateral position reduced positional distress and, in patient 23, provided space between the 11th/12th rib arch and the iliac crest, facilitating visualization of the right kidney.

### Other reasons for difficultly with the prone position

Patient 25 had colon torsion and underwent open sigmoid colon resection and stoma placement. The patient chose the lateral position because the stoma and stoma bag would be compressed in the prone position.

### Efficacy and safety of lateral kidney biopsy

**Figure 5:**
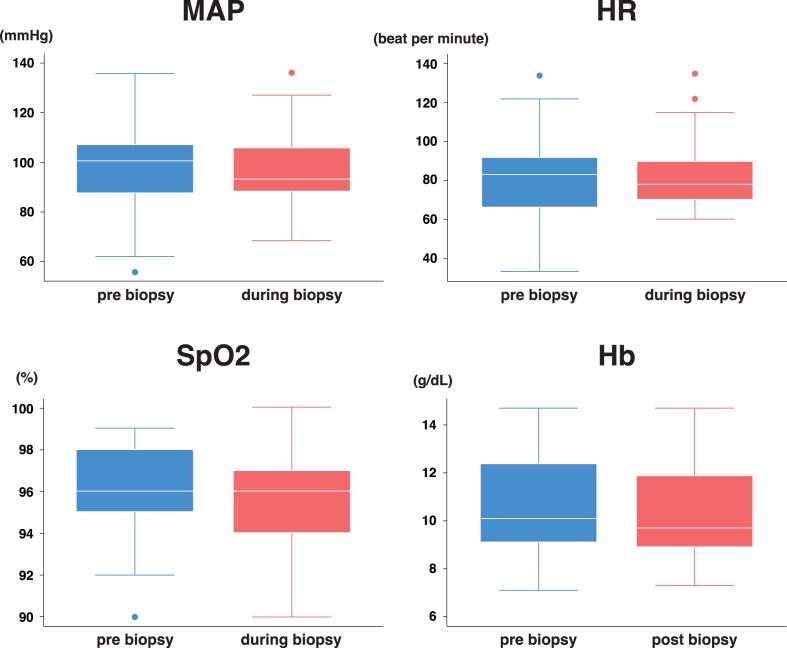
Box plot of changes in vital signs and hemoglobin levels before and after kidney biopsy. The box plot illustrates changes in mean arterial pressure (MAP), heart rate (HR), saturation of percutaneous oxygen (SpO_2_) and hemoglobin (Hb) levels before and after kidney biopsy. No significant differences were observed in any of the variables as determined using the Wilcoxon signed-rank test.

Changes in vital signs pre-, during or post-kidney biopsy are shown in Fig. 5. The mean arterial pressure (*P* = .3967), heart rate (*P* = .2867), saturation of percutaneous oxygen (SpO_2_) (*P* = .3208) and hemoglobin level (*P* = .0639) did not differ significantly before and after biopsy.

**Figure 6:**
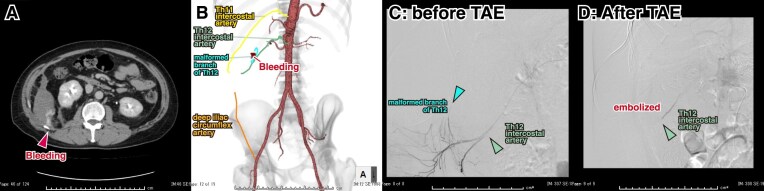
The only case of complication following lateral kidney biopsy (patient 17). **(A)** Retroperitoneal hemorrhage just below the puncture site in the right kidney (arrowhead). **(B)** Three-dimensional arterial mapping revealed a malformed branch of the Th12 intercostal artery (light blue line). **(C)** Angiography before transcatheter arterial embolization (TAE) showed active bleeding from the branch of the Th12 intercostal artery (arrowhead). **(D)** Angiography after TAE with a gelatine sponge showed the successful embolization, with no further visualization of the distal Th12 intercostal artery and cessation of bleeding (arrowhead).

Only one complication occurred among the 25 cases, a retroperitoneal hemorrhage. However, this was not related to the biopsy position. Patient 17 was a 39-year-old man with TAFRO syndrome. He visited his previous physician because of fatigue and jaundice. Examination revealed renal insufficiency, elevated C-reactive protein levels, hepatosplenomegaly and lymphadenopathy. Subsequently, pleural effusion and ascites developed, and TAFRO syndrome was suspected. Glucocorticoid therapy was initiated without a pathological diagnosis and the patient was referred to our hospital for further examination and continued treatment. Owing to dyspnea due to hepatomegaly and splenomegaly, a right kidney biopsy was performed in the left lateral position in the morning. On the afternoon of the same day, severe right back pain developed. Contrast-enhanced CT revealed bleeding from a malformed branch of the 12th intercostal artery. Transcatheter embolization of the distal 12th intercostal artery was performed using a gelatine sponge, which stopped the bleeding and anemia progression (Fig. 6). The above is a complication of accidental contact of the puncture needle with a malformed artery and was unlikely related to the biopsy position.

## Discussion

We performed lateral kidney biopsies in 25 patients who might have otherwise been denied the procedure because of the inability to tolerate the prone position, obtaining adequate tissue for histological diagnosis in all cases. Several notable reports have described kidney biopsies performed in the lateral position. As summarised in Table 3, the published experience with ultrasound-guided native kidney biopsy in the lateral position comprises one prospective randomized study and several observational series/case reports. Chen *et al*. [6] reported that all pregnant women who underwent kidney biopsies in the decubitus position received definitive diagnoses, with complication rates similar to those in non-pregnant women. Gesualdo *et al*. [7] conducted a single-center randomized prospective study and demonstrated the efficacy and safety of kidney biopsy in the spine anterolateral position (SALP). Dugo *et al*. [8] demonstrated that kidney biopsy in the SALP was non-invasive, cost-effective, safe and feasible as a day-to-day procedure for patients with severe obesity and respiratory diseases. In pediatric practice, Tse *et al*. [9] found that lateral positioning under general anesthesia produced biopsy specimens of comparable quality to prone positioning, with similar complication rates, while potentially reducing anesthetic risk and maintaining airway access at all times. A case report also suggested that lateral decubitus may be practical when prone positioning is not feasible owing to pleural effusion and ascites [10]. Compared with these reports, our series expands the clinical contexts in which lateral biopsy may be considered, particularly in patients for whom abdominal compression should be avoided (e.g. AAA) or those with severe cardiorespiratory or musculoskeletal limitations, and highlights additional benefits of lateral kidney biopsy.

**Table 3:** Published reports of kidney biopsy in lateral/modified lateral positions and comparison with the present series.

Author	Year	Design/setting	Patients (indication for lateral position)	Cases, *n*	Position and approach	Needle/guidance	Tissue yield/diagnostic adequacy	Complications	Reference
Chen *et al*.	2001	Retrospective case series	Pregnant women before 30 weeks	15	Decubitus position, lower pole sampling	US guided; 15 Fr Quick-Cut (1990–1991) or 18 Fr automated instrument (1992–1999)	Number of passes, cores and glomeruli were not reported; definitive diagnosis in 100%	Gross hematuria 1/15 (6.7%), resolved within 8 h; no transfusion/intervention	[6]
Gesualdo *et al*.	2008	Single-center prospective randomized study	Obesity (BMI >30) and/or respiratory difficulty in high-risk group (BMI ≤30 and no respiratory difficulty in low-risk group)	110 (45 PP versus 45 low-risk SALP versus 20 high-risk SALP)	SALP (flank elevated ≈30°; access via Petit’s triangle) versus PP	US guided; 16-gauge automatic needle (Monopty™)	Median 2 passes/2 cores, Mean glomeruli/core 15.6 (SALP) versus 15.2 (PP); similar adequacy, no inadequate sampling	Bleeding complications were 9.2 % (SALP) versus 11.8 % (PP); in PP group: 1 AVF requiring chemoembolization	[7]
Tse *et al*.	2013	Retrospective observational comparison	Children under general anesthesia	91 (44 lateral versus 47 PP)	Lateral decubitus under general anesthesia	US guided, biopsy needle Tru-Core II (Angiotech Pharmaceuticals, Vancouver, BC, Canada)	Median 2 passes/2 cores; glomeruli 25 (lateral) versus 30 (prone); sufficient information in all samples to make a clinical diagnosis	Prone group 6%: 3 significant pain, 2 gross hematuria; no intervention, Lateral group 4%: 3 significant pain, 2 gross haematuria, 1 transfusion event	[9]
Dugo *et al*.	2015	Observational comparison	Severe obesity (BMI >30)	222 (27 lateral versus 195 PP)	Spine anterolateral or lateral position, assessing the skin/kidney distance and choosing the position that allowed the shortest path for the cutting needle	US guided, 21-gauge needle (Biopsybell Medical Devices, Mirandola, Italy)	Mean 2 passes/glomeruli 17 ± 3 (LP) versus 17 ± 5 (PP)	No complications reported	[8]
Sathiavageesan	2020	Case report	Pleural effusion and tense ascites	1	Left lateral position through Petit’s triangle	US guided, 18-gauge spring-loaded gun	2 passes, 2 cores; samples adequate for diagnosis	No complications reported	[10]
Present series	2025	Single-center retrospective cohort study	Adults unsuitable for prone position (AAA, respiratory distress, joint pain etc.)	25	Lateral position, puncture posterior to midaxillary line; needle typically perpendicular to back/parallel to floor	US guided, 16-gauge automated biopsy gun	Mean 5.8 passes/4.2 cores; 38 glomeruli, mean glomeruli/core 9.5; no inadequate sampling	1/25 retroperitoneal hemorrhage requiring TAE; not attributed to positioning	

US: ultrasound; BMI: body mass index; PP: prone position; SALP: supine anterolateral position; LP: lateral position; AVF: arteriovenous fistula; TAE: transcatheter arterial embolization.

First, we demonstrated that it could serve as a valuable alternative when prone kidney biopsy poses significant challenges or risks, such as in patients with an AAA, for whom the prone position is contraindicated. In a study of 198 patients with unrepaired AAAs ≥5.5 cm, 112 died, including 45 from probable AAA rupture [11]. The paucity of reports on kidney biopsy in patients with an AAA likely reflects concerns that prone positioning and the associated abdominal pressure might increase rupture risk. We demonstrated that lateral positioning mitigates these risks, enabling safe biopsy procedures in these patients.

We included patients with respiratory distress. Although Gesualdo *et al*. [7] and Dugo *et al*. [8] also reported cases of obesity and respiratory diseases, we included seven patients with massive ascites and pleural effusions, or organomegaly, with TAFRO syndrome as a representative example. TAFRO syndrome is a systemic inflammatory disease characterised by five manifestations: thrombocytopenia, anasarca, fever, renal insufficiency or reticulin fibrosis and organomegaly. In a case series, Mizuno *et al*. [12] reported that the renal pathological finding of TAFRO syndrome was glomerular endotheliopathy, noting that kidney biopsy is often avoided at many institutions because of thrombocytopenia and anasarca-induced respiratory distress. Yoshimura *et al*. [13] reported that kidney biopsy plays a crucial role in diagnosing TAFRO syndrome, especially during relapse, to rule out other inflammatory diseases. Thus lateral kidney biopsy is an effective method for managing patients with TAFRO syndrome.

We also included cases in which the prone position was challenging not only because of obesity but also body shape, such as thinness or scoliosis. In thin patients, limited subcutaneous fat and muscle mass provide little padding, therefore the prone position concentrates the load on bony prominences, such as the sternum, costal margins, iliac crests and pubic ramus. These focal high interface pressures directly cause pain during prolonged contact. Conversely, the lateral decubitus position distributes the load across the shoulder girdle and pelvis and allows targeted offloading of bony prominences with pillows, reducing peak tissue pressure and therefore pain in thin patients. Sawamura *et al*. [14] reported that many patients with rheumatoid arthritis could not undergo kidney biopsies because of significant body deformities, highlighting the feasibility of lateral kidney biopsy in such cases.

**Figure 7:**
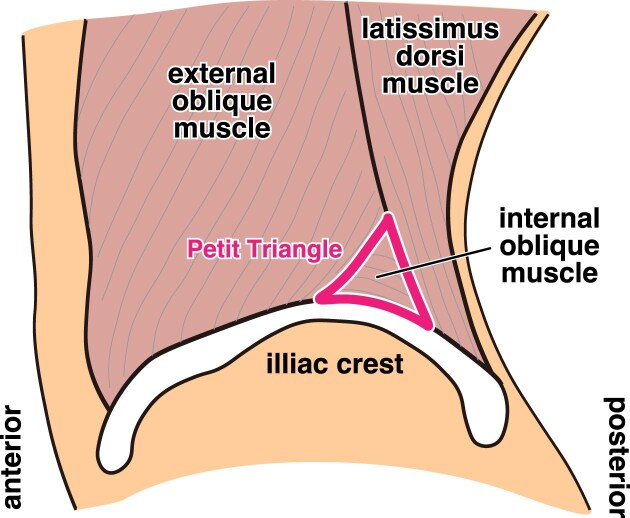
Schematic illustration of the Petit triangle used as the puncture route. It is bound by the latissimus dorsi muscle posteriorly, the external oblique muscle anteriorly and the iliac crest inferiorly, with the internal oblique muscle forming its floor.

Second, proper patient positioning is crucial for a successful lateral kidney biopsy. The purpose of positioning is to keep patients comfortable and create a clear ultrasound image of the kidney. The positioning and puncture techniques described in the Methods section are straightforward. Previous reports have stated that the puncture was performed through the Petit triangle (Fig. 7) (bound by the latissimus dorsi muscle, external oblique muscle and iliac crest) to provide adequate ultrasound visualization of the kidney [7, 10, 15]. However, this approach may require detailed anatomical knowledge, potentially complicating puncture site selection. Our technique differs from the standard kidney biopsy in patient positioning and the requirement to puncture posterior to the midaxillary line.

Third, our report showed that lateral kidney biopsy enabled clinicopathological diagnosis and guided appropriate treatment. Twelve patients had unexpected pathological findings, leading to treatment modifications for eight of them. In other cases, particularly those with TAFRO syndrome, the biopsy provided pathological evidence for diagnosis and enhanced confidence in subsequent treatments. For cases considered high risk for prone-position kidney biopsy, lateral biopsy allows accurate diagnosis, treatment and prognostic assessment.

Conversely, nine patients in our series ultimately received supportive care only after lateral kidney biopsy. In several cases, the histological diagnosis was expected and did not substantially alter the treatment plan. However, biopsy was important for confirming the appropriateness of a conservative strategy, excluding other forms of glomerulonephritis that might require immunosuppressive or other intensive therapies, and providing reliable prognostic information for counselling and preparation for kidney replacement therapy. As a general principle, repeat lateral kidney biopsy would not be planned for these patients based solely on current findings; however, new or worsening proteinuria, haematuria or unexplained changes in kidney function would prompt reconsideration on a case-by-case basis.

The indications for lateral kidney biopsy must be carefully individualized for patients with advanced age or severe comorbidities. In patient 10, a 46-year-old man with Child–Pugh class C cirrhosis, hepatic diabetes and nephrotic syndrome, immunoglobulin A nephropathy (IgAN) was the most likely diagnosis before biopsy. Lateral kidney biopsy confirmed IgAN and showed adverse prognostic features (Oxford classification S1C1). Although immunosuppressive therapy was considered, the risks of glucocorticoid-based treatment in the context of decompensated cirrhosis outweighed potential renal benefits, therefore optimized supportive therapy while preparing for kidney replacement was pursued; the patient eventually progressed to kidney failure and underwent combined liver–kidney transplantation. Patient 11, an 83-year-old man with severe edema and a 17-kg weight gain over 2 months, had an earlier biopsy suggesting possible ANCA-negative vasculitis despite negative serology. Lateral biopsy at our institution revealed collapsing variant focal segmental glomerulosclerosis, allowing discontinuation of glucocorticoid therapy. These examples illustrate that lateral kidney biopsies are reserved for situations in which histology is expected to substantially influence long-term management.

Our study has some limitations. First, it was retrospective and lacked a prone-position control group, as used by Gesualdo *et al*. [7]. We also did not evaluate patient comfort or respiratory distress using a visual analogue scale (VAS), as Gesualdo *et al*. [7] did. However, as they already demonstrated better VAS scores in the SALP group, similar results would likely have been observed regardless of patient background.

Second, we collected more cores and glomeruli than Gesualdo *et al. *[7]; however, the clinical impact of this difference remains unclear. Nevertheless, we believe that our sample size was appropriate. Corwin *et al*. [16] demonstrated that the accuracy of glomerular involvement assessment improves with the number of glomeruli observed, as larger samples narrow the 95% confidence interval for lesion percentages. According to the 2020 Japanese Kidney Biopsy Guidebook, three cores and 20–25 glomeruli are typically required for proper evaluation [5]. Thus our averages of 4.2 cores and 38.6 glomeruli are therefore acceptable. Even under these assumptions, the occurrence of only one unpredictable and not readily preventable complication unlikely related to the lateral position underscores the safety of lateral kidney biopsy. Comparison with core and glomeruli counts from prone-position biopsies remains a topic for future investigation.

Finally, based on the contribution of Mizuno *et al*. [12], our hospital is designated as one of 14 regional core centers for TAFRO syndrome in Japan and therefore receives many referrals of patients with suspected TAFRO syndrome from distant institutions. This background explains the inclusion of several patients with TAFRO syndrome in our series, likely introducing referral bias. Consequently, the relatively high proportion of patients with TAFRO syndrome in our study may not be generalizable to other institutions.

In recent years, alternatives to percutaneous kidney biopsy, including open, laparoscopic and transjugular biopsies, have been actively discussed [17–19]. However, these methods are still under development, have higher complication rates [18, 20] and have not been proven safer than percutaneous biopsies. CT-guided kidney biopsy has been reported as a safe and effective option for selected high-risk patients [21]. Its advantages and limitations differ from those of lateral kidney biopsy. Lateral kidney biopsy enables real-time needle visualization without radiation, can be performed at the bedside and is particularly useful in patients who cannot tolerate the prone position because of respiratory or musculoskeletal problems. Conversely, CT-guided biopsy provides excellent delineation of deep or poorly visualized lesions and surrounding retroperitoneal structures, which is advantageous when targeting focal renal or perirenal lesions; however, it requires transfer to the CT suite and exposure to radiation. Therefore, the choice between lateral ultrasound-guided and CT-guided biopsy should be based on the clinical context and lesion location.

When Iversen and Brun [2] first introduced percutaneous kidney biopsy in 1951, it was performed in the sitting position. Sitting is generally more comfortable than prone, and Amini *et al*. [22] reported sitting kidney biopsy as a feasible option. Applying this concept, we successfully biopsied a perirenal lesion between the liver and upper pole of the kidney by migrating caudally while the patient was in a sitting position [23]. Similar to surgical procedures, selecting the appropriate patient position for kidney biopsy is crucial, depending on the patient’s condition and medical history.

In conclusion, we propose that ultrasound-guided lateral kidney biopsy is a useful alternative method for accurately diagnosing kidney disease in patients who cannot tolerate the prone position.

## Supplementary Material

sfag038_Supplemental_Files

## Data Availability

All data generated or analysed during this study are included in this article. Further inquiries can be directed to the corresponding authors.
